# Frontline Health Workers’ Perspectives of the World Health Organization Skin Neglected Tropical Diseases App in Kenya: Qualitative Study on AI-Embedded mHealth Implementation

**DOI:** 10.2196/81829

**Published:** 2026-07-14

**Authors:** Emily E V Quilter, Ruth Nyangacha, Esther Kinyeru, Aïna Fuster Casanovas, Amberly Brigden, Kenton O'Hara, Diana Atieno, José A Ruiz-Postigo, Carme Carrion

**Affiliations:** 1 Digital Health and Care, University of Bristol, One Cathedral Square, Trinity Street, Bristol, BS1 5TE, United Kingdom, +44 7714250164; 2 Center for Traditional Medicine and Drug Research, Kenyan Medical Research Institute, Nairobi, Kenya; 3 Department for Neglected Tropical Disease, Disease Control and Prevention Cluster, World Health Organization, Nairobi, Kenya; 4 eHealth Lab Research Group, eHealth Center, School of Health Sciences, Universitat de Catalunya, Barcelona, Spain; 5 Vall d'Hebron University Hospital, Institut Català de la Salut, Innovation Unit, Quality, Processes and Innovation Directorate, Barcelona, Spain; 6 School of Computer Science, University of Bristol, Bristol, United Kingdom; 7 Department of Control of Neglected Tropical Diseases, Prevention, Treatment and Care Unit, World Health Organization, Geneva, Switzerland; 8 Department of Medical Sciences, School of Medicine, Universitat de Girona, Girona, Spain

**Keywords:** mHealth, artificial intelligence, clinical decision support, skin neglected tropical diseases, frontline health workers, digital health, Kenya, qualitative research, health systems, World Health Organization, human-AI collaboration

## Abstract

**Background:**

Skin neglected tropical diseases (NTDs) pose significant diagnostic and management challenges in resource-limited settings due to constrained dermatological expertise, frontline health worker (FHW) training, and limited access to diagnostic resources. Mobile health apps with artificial intelligence (AI)–enabled diagnostic imaging capabilities have the potential to enhance clinical decision-making and professional development at the primary care level. The World Health Organization (WHO) skin NTD mobile app uses convolutional neural networks to analyze images of skin lesions and generate differential diagnoses, intended to be used alongside clinical history and examination, to support FHWs in identifying 12 skin NTDs and 24 common skin conditions. Beyond clinical decision support, the app also aims to upskill FHWs in the recognition and management of these diseases. However, the success of such tools depends on understanding users’ needs and the realities of implementation in diverse clinical contexts.

**Objective:**

This study aimed to explore FHWs’ perspectives on the real-world use and impact of the AI-embedded WHO Skin NTDs app on diagnostic workflows, dermatological understanding, clinical decision-making, and FHW-patient interactions across diverse health care delivery settings in Kenya.

**Methods:**

This qualitative study involved 36 FHWs from 5 skin NTD-endemic counties in Kenya. Following a training workshop, FHWs integrated the app into routine clinical workflows from June to October 2024. Data were collected through 15 semistructured interviews (each 30‐45 minutes) and 4 focus group discussions (1‐1.5 hours) exploring FHW experiences across diverse health care delivery contexts. All sessions were audio-recorded, transcribed verbatim, and thematically analyzed using NVivo (QSR International), using a bottom-up inductive coding approach.

**Results:**

FHWs reported that the app facilitated a shift from habitual referral to more proactive case management at the local-level facility, reinforcing clinical ownership and positioning them as local dermatology reference points. It was perceived to enhance diagnostic confidence, strengthen patient trust, and encourage community engagement. Some FHWs described how the app helped mitigate situations for patient stigma due to decreased reliance on public colleague consultations. However, technical limitations (eg, internet dependency and algorithmic errors) constrained consistent use. While most FHWs used the app in line with its intended role as an assistive tool, a minority reported situations of diagnostic deferral to the AI output, highlighting potential considerations of clinical autonomy.

**Conclusions:**

The WHO Skin NTDs app shows strong potential to strengthen frontline dermatological capacity that aligns with WHO strategies to decentralize NTD care and promote “skin health for all.” Our findings underscore the importance of embedding such tools within ethical and pedagogical frameworks that protect clinical autonomy and foster sustainable capacity building. Further research will examine real-world use in situ to guide context-specific governance, ensuring that this AI-embedded tool enhances—rather than displaces—clinical reasoning and epistemic authority.

## Introduction

Neglected tropical diseases (NTDs) are predominantly diseases of those in poverty and impact over 1 billion people worldwide [1]. Skin NTDs (which predominantly affect the skin) constitute a significant subset, accounting for nearly half of all NTDs and disproportionately affect marginalized populations in remote rural areas [2]. The World Health Organization (WHO) has categorized skin NTDs together due to their coendemicity, similar initial symptom presentation, common differential diagnosis, and the potential for integrated multidisease control interventions [1]. Despite their widespread prevalence, skin NTDs are often underreported, resulting in an underestimation of their true prevalence and burden on global health [3].

The consequences of skin NTDs extend beyond physical symptoms. Chronic illness, disability, and visible disfigurement often intersect with stigma, social exclusion, and economic hardship [4,5]. These factors delay care-seeking and contribute to ongoing transmission, disease progression, and long-term disability [6]. In many settings, especially in sub-Saharan Africa, access to specialized dermatological care is minimal—with ratios of 1 dermatologist per 500,000 to 1 million people not uncommon. As a result, frontline health workers (FHWs) often serve as the first and only point of contact for patients with skin conditions [7,8].

While FHWs play a critical role in the early identification and management of skin conditions, most have only minimal formal dermatology training [9,10]. For some of the rarer skin NTDs, this knowledge gap is further compounded by limited opportunities for experiential learning and skill retention [11]. While visual examination remains the most accessible diagnostic method, it requires pattern recognition and contextual clinical judgment—capabilities often undersupported in primary care [12]. Furthermore, advanced diagnostic methods such as polymerase chain reaction and microscopy, the gold-standard for diagnosing many skin NTDs, are often unavailable to FHWs at the grassroots level, where most disease detection and management occur [2,11,13].

To address this gap, the WHO has begun to promote a more integrated approach to skin NTD care through its 2021‐2030 road map, which frames “skin health for all” as foundational to universal health coverage [1]. The strategy emphasizes decentralized care, crosscutting interventions, and capacity-building for FHWs. This agenda was reinforced at the 2025 World Health Assembly where a resolution was adopted recognizing skin diseases as a significant public health concern [14]. Unlike some NTDs managed through mass drug administration, most skin NTDs require individualized diagnosis and longer-term follow-up, increasing the need for clinical decision support at the frontline [2,15]. Enhancing FHW diagnostic dermatological capacity is therefore a strategic priority for reaching skin NTD control and elimination targets [1].

Digital health technologies, particularly mobile health (mHealth) tools, offer promising opportunities to strengthen frontline dermatological care through capacity building [16-18]. More specifically, by embedding artificial intelligence (AI), such as image-based diagnostic algorithms, within these mHealth tools, there is the potential to support FHWs by providing real-time clinical decision support, fostering diagnostic confidence, and facilitating experiential learning in situ [18,19] (in this study, we use this term AI-embedded to refer to mHealth tools that incorporate machine learning models as a defined component of their functionality).

In response to long-standing challenges in the recognition and management of skin conditions at the primary care level, the WHO Skin NTDs app was developed in collaboration with the WHO and No Leprosy Remains [20,21]. The app is designed to assist FHWs in recognizing, documenting, and managing skin NTDs and other common skin conditions, functioning as an assistive clinical decision support tool to enhance clinical diagnostic confidence and capacity [20-22]. The app includes both educational and AI-embedded components, the latter providing image-based differential diagnoses to support clinical reasoning in situ. A more detailed description of the app and its functionality is provided in the Methods section.

However, understanding how such tools are integrated into real-world clinical workflows, and how they may influence clinical reasoning, referral patterns, professional autonomy, and patient experience, is critical. AI-embedded tools in health care can introduce both opportunities and risks, particularly if they shift clinical responsibility or impact human judgment [23]. Evaluating whether such tools are used as intended, and within the boundaries of clinical competence, is therefore central to responsible mHealth innovation and implementation [23]. This study also seeks to examine how the app does or does not support its intended educational use and values, providing evidence to inform its usefulness, and identifying opportunities for iterative design and implementation improvements.

This study explores the use and acceptability of the WHO Skin NTDs app within frontline dermatological care in Kenya, a country with a pluralistic, complex health system and significant gaps in dermatological service provision. Health care delivery in Kenya operates across multiple levels (national referral, county-level, and community-based) [24]. Kenya is endemic for at least 9 skin NTDs including chromoblastomycosis, cutaneous leishmaniasis, post–kala-azar dermal leishmaniasis, leprosy, lymphatic filariasis, mycetoma, scabies, sporotrichosis, and Tungiasis [25,26]. Common skin conditions such as superficial fungal infections are highly prevalent, affecting an estimated 11.57% of the population [27]. Facility-level studies indicate even higher burdens at the point of care; for example, a level 5 hospital in Kiambu County reported eczema in 25.5% of dermatological consultations [28].

This study therefore asks: How do FHWs perceive, interpret, and engage with the AI-embedded component of the WHO Skin NTDs app in real-world clinical settings? Additionally, what do these interactions reveal about the pedagogical, ethical, and systems-level dynamics of deploying AI-embedded clinical decision support tools in primary dermatological care within resource-limited settings?

## Methods

The COREQ (Consolidated Criteria for Reporting Qualitative Research) checklist was used to guide reporting, available in Checklist 1 [29].

### Study Design

This qualitative study explored the experiences of FHWs integrating the WHO Skin NTDs app into routine clinical workflows across 5 counties in Kenya—Baringo, Nakuru, West Pokot, Kwale, and Kajiado, purposefully selected by the Kenyan Ministry of Health due to their endemicity for skin NTDs. Participants represented FHWs across Kenya’s primary health care system, including national referral (level 5), county-level (levels 3 and 4), and community-level (level 2) facilities. Facilities were intentionally sampled across multiple levels of the health system to capture a diversity of clinical and community contexts, resource availability, and workflow structures shaping dermatological care delivery. These levels differ in staffing (eg, nurse-led [level 2] vs clinician-led [levels 3‐5] services), access to diagnostic and treatment resources, opportunities for peer consultation, and referral pathways. The aim of sampling across multiple facility levels and regions in this way was not to support any systematic formal comparison between levels. Rather, sampling across different facilities and regions provided an opportunity for particular distinguishing factors of these varied settings to be invoked by participants, as appropriate, in their explanatory accounts of usage, perceived usefulness, and impact. Data were collected through focus group discussions (FGDs) and semistructured interviews (SSIs) with participating FHWs. This qualitative component forms part of a broader assessment study on the performance of the app’s AI algorithm [30].

### WHO Skin NTDs App

**Figure 1.**
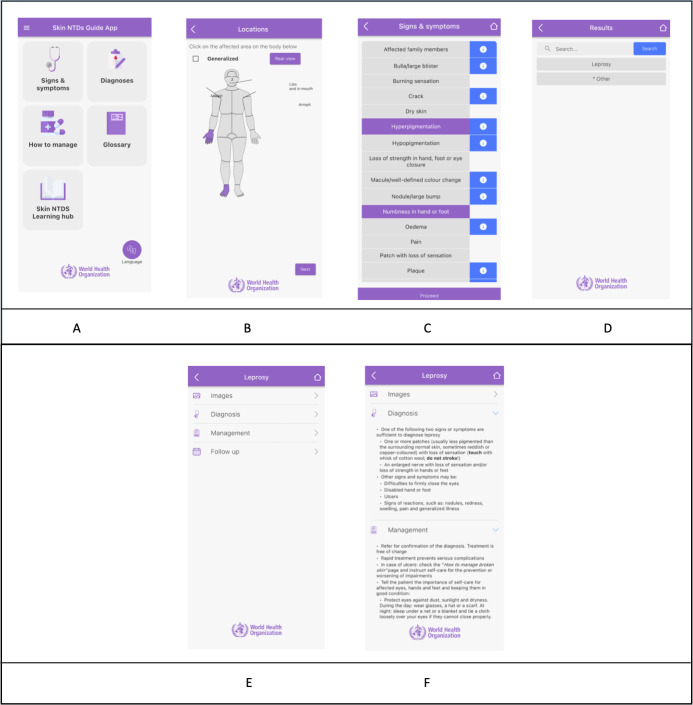
Screenshots from the educational component of the World Health Organization (WHO) Skin NTD app. This component coexists with the artificial intelligence–embedded diagnostic functionality (shown in Figure 2) in the current version of the app. (A) Main home screen of the WHO Skin NTDs app; (B) Body map interface used to indicate the anatomical location of skin lesions; (C) Signs and symptoms interface, where users can select multiple features based on the patient’s clinical presentation (or for exploratory learning outside the clinical encounter); (D) Results screen displaying potential conditions (eg, leprosy) based on the selected lesion location and symptoms; (E) Condition-specific menu (eg, leprosy) displaying sections for images, diagnosis, management, and follow-up; (F) Example educational content page providing clinical information on diagnosis and management. NTDs: Neglected Tropical Diseases.

The WHO Skin NTDs app comprises two integrated components: (1) an educational component providing structured guidance on diagnosis and management, and (2) an AI-embedded component that generates differential diagnoses from uploaded images of skin lesions. The educational component includes interactive skin lesion localization and condition-specific information, clinical images, and management guidance adapted from the WHO training guide on skin NTDs for FHWs (Figure 1) [5]. This can be accessed during or outside clinical consultations to support learning and decision-making.

**Figure 2.**
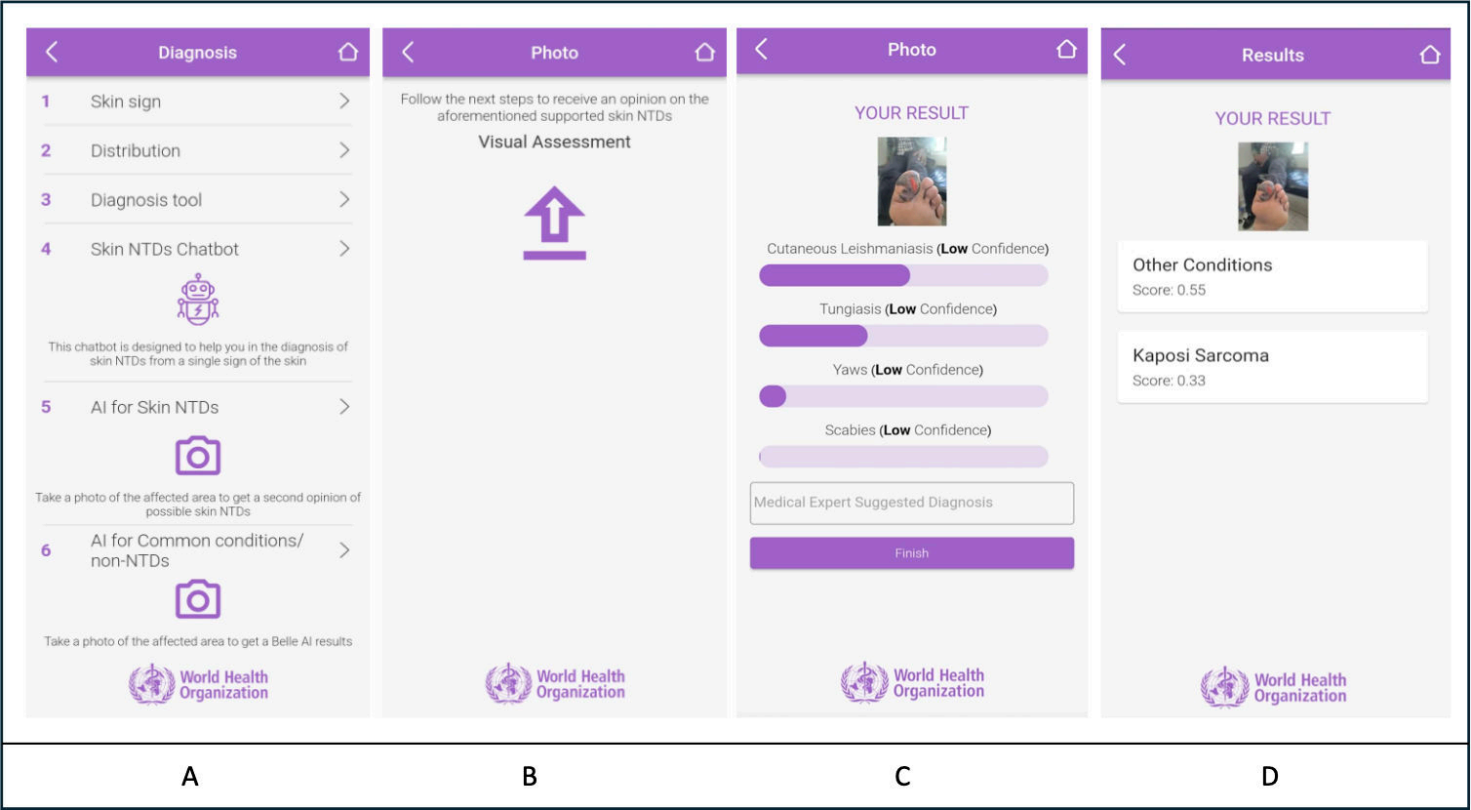
Screenshots from the AI-embedded component of the World Health Organization skin NTD app. (A) Interface of the top-level screen for the AI-embedded component, showing 2 entry points for image upload corresponding to the skin NTD and common skin condition algorithms. (B) Image upload interface for the skin NTD algorithm, where users can capture a photograph within the app or upload an existing image from the device gallery. (C) Example output displaying AI-generated differential diagnoses with associated confidence levels for the skin NTD algorithm. (D) Example output displaying AI-generated differential diagnoses for the common skin condition algorithm; “Other conditions” denotes outputs falling outside the predefined set of 24 common skin conditions included in the model. AI: artificial intelligence; NTDs: Neglected Tropical Diseases.

Within the “Signs & symptoms” component, users first indicate the anatomical location of the lesion using a body map interface (Figure 1A). They then select relevant signs and symptoms from a structured list (Figure 1B and C) and based on these inputs, the app generates a list of potential conditions, which users can explore further to access detailed information on diagnosis and management (Figure 1D-F).

The AI-embedded component is based on convolutional neural network models that analyze and classify uploaded skin lesion images. For each uploaded image, the model generates a small set of differential diagnoses with associated confidence levels (low, medium, and high) across 12 skin NTDs and 24 common skin conditions [21,22] (Figure 2). Given that over 80% of skin conditions encountered by FHWs are common skin conditions, their inclusion is essential for clinical usefulness [31].

The WHO Skin NTDs app is designed to be used during routine patient consultations, although no standardized protocol governs its integration within clinical workflows. In practice, use is flexible and embedded within the consultation itself, typically following initial history taking and physical examination. The anticipated workflow involves FHWs capturing an image of the skin lesion with the phone camera, uploading it to both the skin NTD and the common skin condition algorithms, and reviewing AI-generated differential diagnoses. These outputs are then interpreted alongside patient history and clinical examination findings. The app’s outputs are not prescriptive; rather, they are intended to support clinical reasoning, enabling FHWs to refine or rule out potential conditions. However, given the app’s finite disease library, the true diagnosis may not always be represented.

The algorithm was trained on a dataset comprising 5760 images of skin NTDs alongside 16,577 images of other skin conditions, 2469 images of healthy skin, and 50,000 non–skin images to support classification boundaries [30]. The training set included a diverse range of skin tones, with approximately 70% of images representing dark skin types addressing key concerns around bias in dermatology AI [30,32]. A more detailed account of the app’s design, development process, and training approach is provided elsewhere [30].

### Participants and Recruitment

Participants were nondermatologist FHWs who routinely manage patients with skin NTDs or common skin conditions at the primary care level. Five counties were purposively selected in collaboration with the Ministry of Health, Kenya. Within each county, health facilities and affiliated FHWs were identified with support from the County Departments of Health. From this sampling frame, 50 FHWs were randomly selected and invited by telephone to participate in an initial training and deployment workshop in April 2024.

### Training and Deployment

The training and deployment workshop, held at a centrally located training center in Kenya, introduced participants to the WHO Skin NTDs app. The training combined short didactic sessions with practical exercises. Content included (1) step-by-step instruction on app navigation, image capture, and interpretation of AI-generated differential diagnoses, and (2) guidance on consent practices. Participants engaged in supervised practical sessions where they used the app on peers to familiarize themselves with consent practices, image capture, and result interpretation.

Following the first training workshop, participants were added to a WhatsApp group with members of the study team to facilitate ongoing technical support and troubleshooting during the deployment period. Participants were invited to integrate the app into their routine clinical practice over a deployment period of approximately 6 months. After the 6-month deployment period, a second evaluation session was convened in November 2024, again at a central location in Kenya. This session focused on understanding participant reflections on their experience using the app and comprised the main qualitative data collection activities. During this session, FGDs and SSIs were conducted to explore participants’ experiences using the app, including perceived benefits, challenges, and contextual influences on use. Of the original 50 FHWs invited, 47 attended the first training workshop and participated in the deployment phase. Thirty-six attended the second evaluation workshop and contributed to the qualitative data collection.

### Ethical Considerations

This study was approved by the research ethics committee at the Open University of Catalonia, with the Kenyan Medical Research Institute (KEMRI) protocol number KEMRI/SERU/CTMDR/122/4959, Open University of Catalonia reference number CE23-RC21, and the Kenya National Commission for Science, Technology and Innovation license number NACOSTI/P/24/35733. Written informed consent was obtained from all participating FHWs. The FHWs were informed that participation was voluntary and that they could withdraw from the study at any time without any consequences. All FHWs participating in the second workshop provided written consent for audio recording and the use of data for analysis and publication. In addition, FHWs obtained written informed consent from patients before using the WHO Skin NTDs app during clinical consultations during the 6-month study period. Patients were informed that participation was voluntary and that their standard of care would not be affected by refusal to participate. All data were deidentified prior to analysis to ensure participant confidentiality. No personally identifiable information is reported in this study. FHWs were not financially compensated for participating in this study, although travel and subsistence costs (11,000 Kenyan Shillings [1 Kenyan Shilling=US $0.0077 as of June 26, 2026] per FHW, based on government rates) were provided to support workshop attendance. Mobile data bundles (5000 Kenyan Shillings per FHW) were also provided to support app use within clinical workflow.

### Data Collection

Data were collected through 15 SSIs (each lasting 30‐45 minutes) and 4 FGDs (1‐1.5 hours, 8‐10 FHW in each). The SSIs enabled a focused exploration of individual FHW perspectives, while the FGDs offered opportunity to encourage debate and consensus building, highlighting areas of agreement and contrast in perspectives on the WHO Skin NTDs app use within diverse clinical workflows. The interviewers (EQ, RN, CC, AF, and DA) used semistructured topic guides to guide FGDs and SSIs, which were reviewed by all authors prior to the workshop (MultimediaAppendices 1  2). All FHWs participated in FGDs, and 15 FHWs were purposively selected to participate in SSIs to ensure a balanced mix across counties.

Both SSIs and FGDs were used to enable complementary insights. SSIs allowed for in-depth exploration of personal experiences, with participants asked to recount specific instances of app use in situ. This yielded rich, contextualized accounts of how the app was integrated into individual diagnostic decision-making, including nuanced challenges and variations in use. FGDs, by contrast, enabled group-level reflection, providing a space to examine shared practices, compare perspectives, and identify points of consensus or disagreement regarding the app’s usefulness role in workflow and broader systemic challenges. This combination of methods was intended to enhance both the depth and breadth of understanding regarding app integration in routine care.

All interviews and FGDs were conducted in English (an official language in Kenya). Each FGD was cofacilitated by a local research scientist from KEMRI (RN and DA) and a researcher from the Global North (EEVQ and CC). All FGDs and SSIs were recorded on portable Dictaphones and stored on a secured university-licensed cloud service (OneDrive). Field notes were taken by all researchers during and after both FGDs and SSIs and were used to support the thematic analysis.

### Data Analysis

Audio recordings were transcribed verbatim by the corresponding author (EEVQ), supporting deep familiarization with the data. Transcripts were analyzed thematically using NVivo (version 14; QSR International) software. Given that this is the first qualitative assessment of the AI component of the WHO Skin NTDs app usability and acceptability, a bottom-up inductive coding approach was selected to support the iterative construction of themes grounded in participant narratives. This exploratory approach aimed to capture nuanced insights that may inform the application of appropriate theoretical frameworks in future studies, as the evidence base on the app’s integration expands. EEVQ conducted initial coding of all transcripts. A subset of transcripts was independently double coded by AB and KO. Coding discrepancies were resolved through team discussion, and the codebook was iteratively refined. The full dataset was analyzed using reflexive thematic analysis, with codes inductively organized into 4 higher-order themes within the research team [33].

The derived themes from the analysis made reference to behavioral patterns and interdependencies that lent themselves to communication through systems-thinking visualizations. With this in mind, we constructed 2 causal loop diagrams (CLDs) grounded in FHW narratives to provide a structured way to visualize how system-level dermatological workflow factors interact in practice, both before and after app integration [34]. The intention here is not to generate formal theory but rather to offer descriptive representations of diagnostic and referral pathways and contextual factors shaping their progression as revealed through the FHW accounts. In this respect, the CLDs function as an analytic and communication tool to scaffold the interpretation and presentation of the overall thematic narrative.

## Results

### Overview

The 36 participants who attended the second workshop in November 2024 comprised FHWs from 5 counties in Kenya: Nakuru (n=11), a fairly urbanized county situated in the Rift Valley; Baringo (n=8), also situated in the Rift Valley, characterized by semiurban and rural areas; Kajiado (n=6), bordering Tanzania in southern Kenya with a seminomadic population; West Pokot (n=8), a remote rural county bordering Uganda, predominantly inhabited by nomadic pastoralist communities; and Kwale (n=3), a largely rural, coastal county in southeastern Kenya [35-39]. Participant characteristics are described in Table 1.

**Table 1.** Participant details: ID, county, job role, and level of health care facility (specific county names have been removed to protect participant anonymity).

Participant ID	Job role	Level of health care facility
1	Public health officer	Level 5
2	Nurse manager	Coordinator
3	Clinical officer	Level 4
4	Clinician	Level 4
5	Clinician	Level 5
6	Clinician	Level 5
7	Clinician	Level 5
8	Clinician	Level 5
9	Nurse	Level 5
10	Clinician	Level 4
11	Clinical officer	Level 4
12	Clinician	Level 4
13	Clinical officer	Level 5
14	Nurse	Level 5
15	Community health nurse	Level 2
16	Nurse	Level 2
17	Public health officer	Level 3
18	Clinician	Level 5
19	Clinician	Level 2
20	Clinician	Level 2
21	Clinician	Level 4
22	Clinical officer	Level 3
23	Clinical officer	Level 3
24	Nurse	Level 2
25	Clinical officer	Level 5
26	Clinical officer	Level 5
27	Doctor	Level 4
28	Clinical officer	Level 4
29	Nurse	Level 2
30	Clinical officer	Level 5
31	Clinical officer	Level 5
32	Clinician	Level 5
33	Clinician	Level 2
34	Pharmacy technician	Level 3
35	Clinical officer	Level 4
36	Clinician	Coordinator

**Figure 3.**
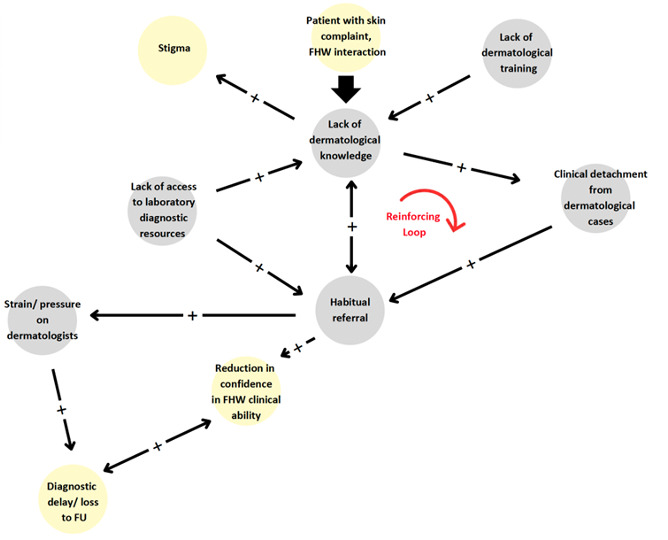
Causal loop diagram: preapp context, reported systemic challenges in dermatological care. FHW: frontline health worker; FU: follow-up.

**Figure 4.**
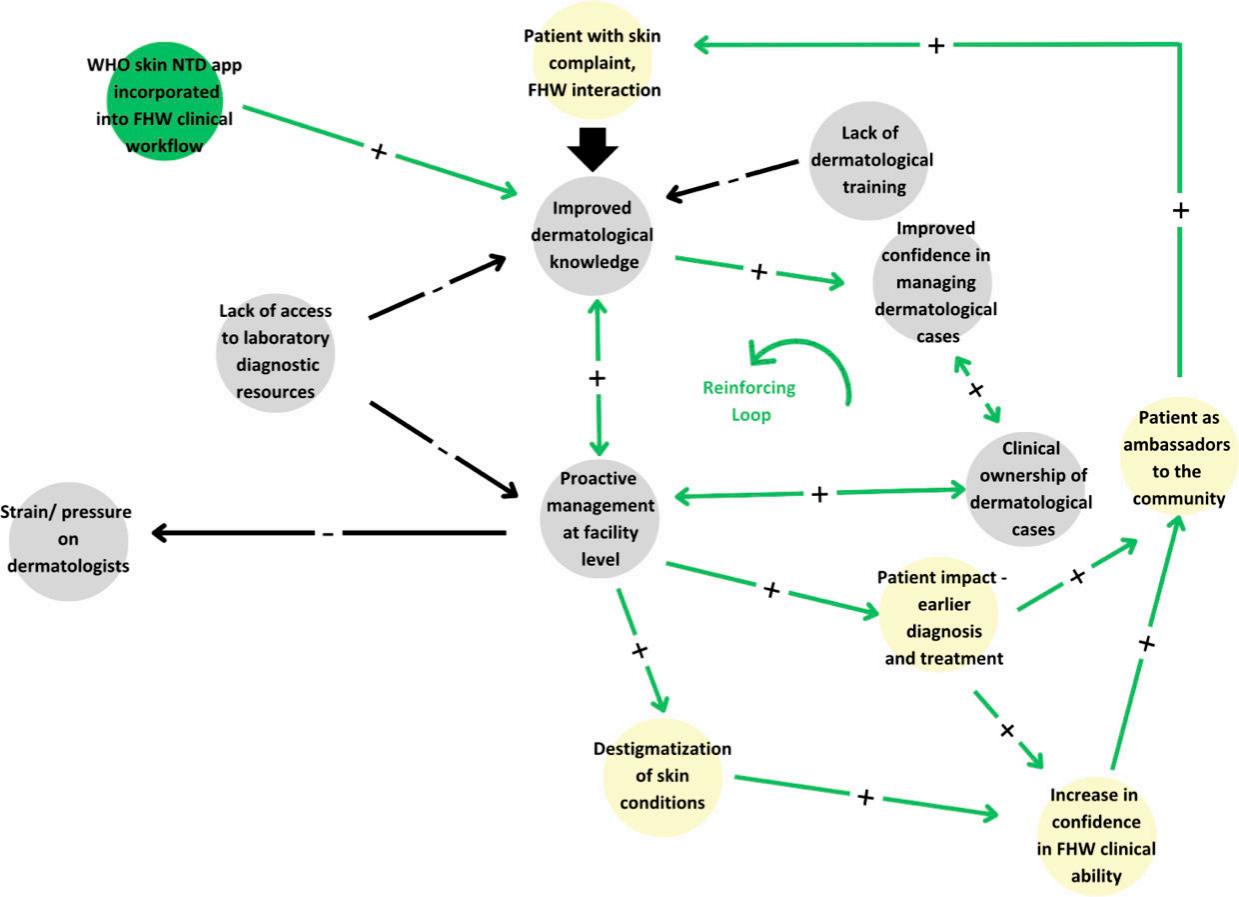
Causal loop diagram: postapp integration and shifts in clinical workflow. FHW: frontline health worker; NTD: Neglected Tropical Diseases; WHO: World Health Organization.

The thematic analysis highlighted shifts in dermatological care delivery, representing the complex dynamics before and after app integration, visualized through 2 CLDs (Figures 3 and 4). The CLDs are grounded in FHW narratives, with illustrative quotes supporting each node (Multimedia Appendix 3). Yellow nodes represent patient-related factors, gray nodes denote FHW-related elements, green arrows indicate changes postapp implementation, and reinforcing loops perpetuate challenges or amplify improvements. Connections depict the relationships between these factors, as articulated by FHWs, indicating whether they have a positive or negative influence. The diagrams synthesize FHW perspectives across multiple facility levels rather than disaggregated by facility levels. However, we acknowledge that the key elements of aggregated narratives will have a differential impact across the different facility.

Before app integration, FHWs reported patterns of habitual referral of dermatological cases, where nonspecialist dermatological knowledge often limited immediate diagnostic assessment capability, combined with limited access to diagnostic resources and specialist training that would enable necessary upskilling (Figure 3). This resulted in FHWs expressing a sense of clinical detachment, reinforcing a cycle where FHWs avoided managing dermatological cases at their local facility; instead, depending upon habitual referral to higher-level facilities and specialist services. FHWs expressed that this referral dependency strained dermatology services, which they associated with diagnostic delay and subsequent patient attrition (loss to follow-up). The diagram further highlights a consequent reinforcing feedback loop—the more FHWs referred cases, the less experience they gained in managing them, further diminishing confidence and knowledge in dermatological care.

After app integration, FHWs described a shift toward more immediate dermatological case management at their local-level facility, interrupting the cycle of habitual referral to higher-level facilities and specialist services (Figure 4). The app was perceived as enhancing dermatological knowledge acquisition and development, reinforcing a sense of clinical confidence and greater feeling of ownership of dermatological cases. Local facility case management was perceived to facilitate earlier diagnosis and treatment, which FHWs linked to improved patient trust in their clinical abilities. Additionally, FHWs highlighted the app’s role in destigmatization of skin conditions, as greater diagnostic confidence reduced the need for voyeurism of collaborative deliberation over patients. FHWs spoke of patients becoming “ambassadors” within communities, actively promoting care-seeking behavior based on their positive experiences with the app. The diagram further highlights a consequent reinforcing feedback loop—as FHWs gained dermatological knowledge, their confidence in managing cases increased, fostering greater clinical ownership. This, in turn, reduced reliance on specialist referrals as FHWs managed more cases at the facility level, with potential implications for strengthened primary dermatological care.

These system-level causal-loop representations (Figures 3 and 4) offer high-level synthesis of FHW-identified factors influencing the transformation of care and referral pathways. Building on this, we present a deeper dive into the phenomenological accounts of FHW-reported app use and the perceived transformation of practice experienced arising from its adoption.

### From Habitual Referral to Local Facility–Based Ownership of Dermatological Cases

The integration of the WHO Skin NTDs app into diagnostic workflows was seen to facilitate a shift from habitual referral to local facility management. FHWs described this transition as improving patient retention and fostering wider community engagement (Figures 3 and 4).

#### Pre - app: Clinical Detachment and a Clinical Model of Habitual Onward Referral

Before the introduction of the app, FHWs described a sense of clinical detachment from dermatological patients, often perceiving these cases as outside their professional remit (Figure 3). This detachment was largely attributed to “very little knowledge about skin or maybe dermatology*”* [Participant 23, clinical officer, level 3].

This lack of dermatological expertise seemed to contribute to a “clearing and referring” culture in which dermatological cases were routinely categorized as “clearly not for me” [Participant 3]. Consequently, patients were often referred to higher-level facilities and specialist services without comprehensive clinical assessment (Figure 3). High patient volumes further exacerbated these practices whereby referral, in lieu of clinical knowledge, contributed to a more efficient throughput to help manage overall patient load: “sometimes 120, 130, 150 [patients a day]...you have a lot of patients to attend, it’s not only the skin ones” [Participant 13, clinical officer, level 5]. Collectively, FHWs spoke of viewing dermatological conditions as inherently complex and firmly within the domain of specialist dermatologists, reinforcing a reliance on specialist referral as a default rather than a considered clinical choice:

*The patient with skin NTDs...we didn’t want much to know much about it since we didn’t know how to make the diagnosis...we didn’t want to learn...It was difficult to make diagnosis and in terms of management, it was still difficult for us to manage those conditions...we were not in a position to make that diagnosis...These were conditions that were supposed to be managed by specialist. Specialised people, not by us. Yeah, ours was clearing* [patients] *and referring...and we wouldn’t have followed up.*

[Participant 3, clinical officer, level 4]

Notably, some FHWs framed habitual referrals not as an inability to manage but as a need for diagnostic “*confirmation*” [Participant 23], highlighting the potential role of confidence in enabling local facility–based management.

#### Post - app: The App’s Perceived Role in Shifting Toward Clinical Ownership and Local Facility Management

FHWs perceived that the WHO Skin NTDs app disrupted habitual referral patterns by providing clinical decision support, reframing dermatological conditions as more manageable within primary care. Where appropriate, this shift seemed to encourage more immediate and confident management at the local-level facility (Figure 4):

*We used to refer all patients with skin conditions. But now with the app, we try to manage the patients without sending all of them to the dermatologist.*

[Participant 32, clinician, level 5]

Many of the health workers described a developing sense of clinical ownership, actively positioning themselves as local dermatological experts with colleagues now seeking their consultation for dermatological cases:

*From the time we started using this app, it has, really had a positive impact. Because in our health facility, if anyone comes across a patient with skin lesion, he or she will first send the patient to me. Even if I am off, they will call me...“come and see there is a patient with a skin lesion, come and use the app, we see what is the problem.”*

[Participant 21, clinician, level 4]

#### Impact of Local Facility Management on Patient Retention and Engagement in the Full Illness Trajectory

Before the introduction of the app, FHWs described how their habitual referral patterns would “delay the management or the treatment of this patient.” The reasons for this are that the specialist dermatology clinics to which the patients were being referred were infrequent, happening maybe “once in a month or once in a week” [Participant 13, clinical officer, level 5]. As such, patients would be required to wait until the clinics were available and take further time out to travel. In response, many FHWs (notably from level 2 and 3 facilities) described how they informally adopted WhatsApp to enable a limited form of remote dermatological consultations as a workaround. These kinds of practices point to the broader informal integration of mobile phones into everyday health care delivery—an improvised form of telehealth that enables more immediate access to specialist input without requiring patients to travel—in principle, easing the burden of referral. However, FHWs also highlighted the fragility of this workaround, particularly when “sometimes they [the dermatologist] don’t even reply” [Participant 19]. Reliance on asynchronous responses from often-unavailable specialists was therefore seen, at times, as a potential source of delay in patient management:

*OK, I think it* [WHO skin NTD app] *also saves time since like people who are in a rural facility, when they come across a skin disease and they don’t have knowledge about it, they call a dermatologist. And the dermatologist says “take a photo and send it through Whatsapp.” So it takes time to get the results. But with the app, it helps diagnosis and you get the patient directly into the system.*

[Participant 34, pharmacy technician, level 3]

FHWs widely reported that the app’s immediacy of decision support was seen as important in enabling earlier treatment at the local-level facility, reducing the reliance on informal WhatsApp workarounds (Figure 4). Significantly, the subsequent reduction in assessment and treatment delays was felt to be further beneficial in reducing the potential health complications associated with treatment delay:

*It* [WHO skin NTD app] *is a key app for any primary healthcare worker, because one, it’s going to help in giving the treatment to the patients...it’s going to stop delay in giving the patient treatment. And that will reduce the complication associated with each particular condition depending on each condition you are dealing with. “Cause as we delay, there are problems of that condition.”*

[Participant 18, clinician, level 5]

Managing dermatological patients at the local-level facility was widely reported as “cost-effective for our clients” [Participant 2], reducing both direct costs (eg, transport and consultation fees) and indirect costs (eg, loss of earnings due to travel and appointment wait times). FHWs linked these cost savings to improved patient retention, with fewer patients “defaulting” from care [Participant 2]:

*The impact to my patient...it has helped so much because it will reduce the cost because generally rather than the patient having to go and look for a dermatologist in Nairobi or in another place, you are able to make that diagnosis* [at the facility level] *and help that patient.*

[Participant 13, clinical officer, level 5]

Some FHWs also reported a sense that patient confidence in local facility–based care improved with app use. They described that for some patients, seeing the app used in their consultation through digital imaging of their skin lesion appeared to foster “a lot of hopes—that maybe here the treatment will come*...”* [Participant 9, nurse, level 5]. However, there were also reports that some patients remained skeptical of the app, particularly those with prior experiences of misdiagnosis. This skepticism was often expressed as hesitancy around image capture. As one FHW described: *“*One [patient with a skin condition] was saying ‘I have been facing several dermatologists without any improvement, so why would I assist you with consent to take a photo from part of my body?’” [Participant 25, clinical officer, level 5]. Similarly, others described that prolonged illness trajectory appeared to shape patient perceptions of the app’s value “*s*omeone has maybe a lesion for a very long time, then...‘here you are, you want to take a photo of that lesion. So, what difference will it make?’” [Participant 22, clinical officer, level 3].

#### Impact of More Immediate Local Facility Management on Community Engagement

Multiple FHWs described an unexpected outcome of earlier facility-level management. Having experienced the app’s role in clinical decision support, FHWs reported that patients then encouraged others to seek similar care, emerging as informal community “ambassadors*”* [Participant 25], seemingly facilitating peer-driven health-seeking behavior (Figure 4):

*And also to the community, once you use the app in diagnosing one patient, he or she will go on, referring another one, “go to the facility, the nurse will take a photo.”*

[Participant 29, nurse, level 2]

FHW associated this community advocacy with *“*hope,*”* driven by the belief “that there is something that will come out of that [the app]*”* [Participant 24, nurse, level 2].

### From Limited Dermatological Knowledge to Perceptions of Increased Knowledge and Confidence in Case Management

Findings suggest that the shift from habitual referral to local facility case management was driven by FHWs’ perception of improved dermatological knowledge, in turn enhancing clinical confidence (Figures 3 and 4). The majority of FHWs suggested that the app did not replace clinical judgment but seemed to reinforce it—functioning as an assistive tool that encouraged active engagement with differential dermatological diagnoses.

#### Knowledge Acquisition and Development Through Diagnostic Comparison and Self-Directed Education

The app’s provision of differential diagnoses appeared to encourage curiosity, with many FHWs describing how this broadened their clinical awareness of skin conditions (Figure 4). Encountering unfamiliar or unexpected diagnostic suggestions seemed to prompt more active clinical reasoning, including questioning, comparison, and reflection on local disease patterns. As one FHW described:

*...it has opened my mind more, giving me more learning on a lot of exposure to many conditions...you take a photo, it says Yaws, and Yaws is not common in that region. So you get suspicious...why? So you have to ask several questions...*.

[Participant 13, clinical officer, level 5]

FHWs also described engaging in self-directed learning in response to these prompts, seeking additional information through books and online resources when presented with unfamiliar conditions. One participant noted that the app “challenged [their] knowledge” and motivated them *“*to go and read...to understand more about the disease*”* [Participant 33, clinician, level 2].

Beyond knowledge acquisition, FHWs noted a deliberate process of comparing the app’s differential diagnosis— referred to by some as *“*impressions*”* [Participant 3]—with their own clinical assessments. This comparative process involved integrating patient history, physical examination, and app output to support diagnostic reasoning: “...once I ask the app what is their opinion, it’s it will help me see if my clinical judgement is next to what the app is actually saying*”* [Participant 18, clinician, level 5]. FHWs emphasized how this process enabled them to refine, rather than replace, their clinical judgment. The app was described as providing “room” to rule out or reconsider working diagnoses based on likelihood and clinical context [Participant 21].

While the app was widely perceived as supportive, most FHWs recognized that its outputs were not infallible. As one participant noted, it should be used as “support...and then you add to the knowledge you have*”* [Participant 19]. However, engagement with the app varied, with a minority of FHWs expressing high levels of reliance on its outputs, at times treating them as definitive and “100% this is the skin condition*”* [Participant 1, public health officer, level 5]. In cases of diagnostic discrepancy, FHWs reported prioritizing peer consultation over defaulting to the app’s output, emphasizing the continued importance of clinical judgment and collaborative decision-making. As one FHW reflected: “You have to still have your own clinical judgment...If you don’t agree [with the app output]...you must consult*”* [Participant 6].

#### Confidence Gains as an Outcome of Incremental Knowledge Acquisition and Development

FHWs reported their “confidence building up” in dermatological case management as they perceived development in their dermatological knowledge through continued app use [Participant 19, clinician, level 2].

*So it* [the app] *has impacted me positively because now I’m very confident to tackle any skin condition rather than before. Before seeing that patient, you can see the patient from far and then you are thinking me how am I going to manage this patient now? Where can I start, from what you see? But when this app came, you have that confidence to see that patient and come up with a diagnosis.*

[Participant 11, clinical officer, level 4]

Importantly, this confidence was not derived from blind reliance on the app’s output but from the alignment between the app’s suggestions and FHWs’ clinical judgment. When the app’s diagnostic suggestions corroborated their own clinical assessments, the majority of FHWs reported an increased “morale*”* and motivation to engage more deeply with patient management:

*The positive part is that when you come into contact with a client, and then you use the app, and the app says the exact diagnosis you have been thinking of clinically and then you feel comfortable in managing the patient. And then that morale of doing more for the patient comes in.*

[Participant 12, clinician, level 4]

This confidence was further reinforced by the app’s availability as a “backup*”* [Participant 23] tool, particularly in settings where dermatological expertise and resources were scarce:

*If I have something that I’m not very sure, I’m not worried or I’m not anxious because I know I have the app, which is a tool that can assist me... I’m confident because it’s like knowing there’s somebody here who can, you can always consult and refer to...I’m sure that there’s somebody who can help me. And that’s somebody is the app*.

[Participant 23, clinical officer, level 3] (Figure 3).

#### Reduced Dependency on Public Deliberation Increases Patient Trust and Alleviates Stigmatization of Patient as a Clinical Spectacle

Before app integration, FHWs reported that one consequence of limited dermatological knowledge was the felt need to call multiple colleagues, “maybe even 2-3 people to come to the same room and discuss the patient” [Participant 13]. While perhaps intended as a means of clinical support, many FHWs reflected on how this public deliberation created awkward and uncomfortable situations for patients, reinforcing stigma around dermatological conditions (Figure 3):

*Before, every time they* [the patient] *comes, we call each other “come confirm if this is leishmaniasis, come confirm if this is leish”...By the time you are calling more people to come and see, you see, the patient feels stigmatised.*

[Participant 2, nurse manager, coordinator]

Beyond stigma, FHWs observed that patients often perceived these public deliberations—conducted in front of the patient (and distinct from standard peer consultation outside the consultation space)—as a sign of clinical uncertainty, reducing trust in their ability to provide appropriate care. Participant 2 elaborated:

*...if a patient comes in and you are not sure of the diagnosis, the patient may feel that this clinician is not confident. Why has the clinician have to call others to come and verify the diagnosis, it means that the clinician is not confident enough...even the clinician doesn’t know what I’m suffering from. They will not even feel confident with the treatment that you write for them*.

However, with the app as “a partner that can help you make a diagnosis,” FHWs described an increased confidence to make clinical decisions more independently, “instead of calling a colleague, several clinicians to come” [Participant 15]. FHWs reported that this shift contributed to a reduction in stigma, as patients were no longer subjected to group deliberations during diagnosis:

*Before we get hold of this app, when you see a condition where you suspect this is an NTD, you have to call your colleague, maybe even 2‐3 people to come on to come to the same room and discuss the patient. So, for now at least, there is that reduction in the stigma because you are only with a patient. So, there is a lot of confidentiality.*

[Participant 13, clinical officer, level 5]

#### Opportunities to Extend Knowledge Development Beyond Clinical Decision Support to Full Illness Trajectory

While the app was reported to support dermatological learning and diagnostic reasoning, many FHWs reported that it “is limiting in terms of management… it does not give us information on treatment.*”* This limitation added complexity to clinical workflows and often required FHWs to “use the app alongside Google...to find how to treat” [Participant 8, clinician, level 5]. Although the app does contain management guidance in a separate section, FHWs envisioned embedding treatment and management guidance directly after diagnostic suggestion to help reinforce incremental learning across the full illness trajectory. Participant 8 went on to explain: “...it would be better if, for the purpose of frontline workers who really don’t have a lot of knowledge in dermatology, every information comes to the app so that once you have said that you have uploaded the photo, you get a diagnosis, get treatment.”

### The WHO Skin NTDs App Facilitating Patient Involvement in the Diagnostic Process

Findings show that the integration of the WHO Skin NTDs app into clinical workflows appeared to shift diagnosis from a clinician-driven process to a more participatory model, actively involving patients in their care. Initial narratives from FHWs indicate that the app seemed to function as both a clinical decision support tool and a patient engagement mechanism.

#### Strengthening Patient Involvement Through Diagnostic Transparency and Participation

FHWs described how, for some patients, seeing the app’s interface and diagnostic suggestion appeared to reinforce engagement in their own care:

*This app, when I used it on my patients, they were a bit happy because when I showed them the diagnosis* [on the app], *we could share together. So, most of them were actually happy because of the technology.*

[Participant 5, clinician, level 5]

The app also appeared to promote more interactive consultations, with some patients becoming increasingly engaged in discussions about their skin conditions and expressing curiosity about the diagnostic process:

*This time around when you are using the app you explain to the patient more and also the patient was curious to know more about, about the app and sometimes also they were curious about the skin diseases. Maybe they could ask you, “have you ever seen maybe a skin disease like this with anyone else, or is it? Or is it me alone? So, there was a lot of discussion between the patient and me now. They’ll tell you...“this time around the way you have explained to me, I think I’ll get well*.*”*

[Participant 22, clinical officer, level 3]

Beyond sharing diagnostic suggestions, FHWs felt facilitated by the app in engaging patients in real-time diagnostics, making consultations more collaborative. In turn, FHWs attributed this increased participation with strengthened patient confidence:

Participant 3 [clinical officer, level 4]: *...the best thing is to involve your patient in the* [diagnostic] *process.*

Interviewer: *And the app allows you to do that?*

Participant 3: *Of course, whenever you’re using it, it is our phone. You know, the way we sit together, the way we sit with our patients in our facility. My patient will come. OK we sit at an angle we normally use a system* [computer system], *this patient wherever he’s sitting he will have to see the system...It’s almost next to each other...So, the patient can see everything you’re doing...so at least the patient will follow the process, and by the end of the day, he’ll see the diagnosis that you get from the app. So, it is still build confidence in this patien*t.

#### Technical Limitations and Their Impact on Patient Involvement in the Diagnostic Process

FHWs described varied experiences with internet connectivity, which shaped their ability to use the app in real time and, in turn, influenced the participatory nature of consultations (the AI-embedded component of the app currently functions online only). Some reported no issues “in terms of connectivity” [Participant 3], while others anticipated connectivity issues but developed workarounds such as identifying areas with stronger signal:

*My first impression was since the app is using data bundles, and connectivity in my area is a bit poor. So, I thought it would give me a really hard time uploading the photos, which wasn’t the case so much because I designated an area that was a bit faster.*

[Participant 24, nurse, level 2]

However, in more rural settings, FHWs described network dependence as a major limitation, preventing the app from being used consistently: “I think the challenging part of this app is the problems when there is no network...so you cannot use the app... in rural facility...you cannot assist the patient using the same app” [Participant 12, clinician, level 4].

Beyond connectivity, specific technical failures disrupted patient involvement more directly. One recurrent issue was the unsettling error message “not human skin” [Participant 36], which appeared during attempts to capture images in live consultations. Despite multiple adjustments, this issue often persisted: “The first time I got the response of not a human skin. I kept on trying several times, like increasing the light, maybe the light was low, changing postures...I tried several times, but still it failed” [Participant 32, clinician, level 5]. Such breakdowns seemed to introduce moments of uncertainty during consultations, undermining both interactional flow and patient confidence. As one FHW reflected:

*The problem of course is when the patient looks at you it also gives stigma to those patients. Including myself...you are wondering “what I am doing, am I doing the right thing.” It’s the patient and me, and the patient trusts me, and I look like this thing is not working then the patient also thinks ‘he does not know what he is doing, using this app*.

[Participant 1, public health officer, level 5]

In some accounts, some FHWs attributed this error message to the technology’s failure to recognize darker skin tones:

*Another disadvantage as part of it is when you take a photo, especially in our place, you see the African skin, the app will tell you that’s not a human skin.*

[Participant 12, clinician, level 4]

### Interconnectedness**:** Expanding the Role of the WHO Skin NTDs App

FHWs envisioned the WHO Skin NTDs app evolving beyond its current role as an assistive tool for capacity building within the individual clinical encounter. Their recommendations highlighted its potential to function as part of an interconnected health system, linking clinical dermatological care with broader health system infrastructure.

#### Connecting Clinical Services and Integration Into Local Digital Infrastructure

The majority of FHWs emphasized the importance of embedding the app within existing digital health systems, particularly Kenya’s electronic medical record (EMR), to streamline clinical workflows and facilitate referrals. Integration was viewed as important for both sustainability and routine care.

*Using the computer system* [EMR], *it could improve the referral system for so many clients from skin NTDs to the facilities. So, there maybe should be a connection between the app in their phone and the gadget in the facility*.

[Participant 17, public health officer, level 3]

*The Kenya Government is encouraging the digitalisation of the health system...So, any system which is coming, any digital app which is coming, it has to be interoperable with those systems of government* [Kenyan EMR]. *Those ones, it’s just helping in terms of data management, not in terms of diagnosis.*

[Participant 10, clinician, level 4]

These insights highlight a potential opportunity for additional interoperability, with the app positioned not as a stand-alone tool but as part of an integrated digital ecosystem supporting sustainable service delivery.

#### Connecting Clinical Services to Broader Public Health

FHWs commented how greater interoperability could potentially facilitate closer alignment with national health systems in ways that could further benefit broader public health. Beyond point-of-care decision support, the app was imagined as a tool for real-time disease surveillance, resource mobilization, and data-informed planning.

*It* [the app] *will also help in resource mobilisation. Because at the community level, at the lower level, level 1, level 2, level 3, you will be able to know those diagnosis using the app and the clinician judgment. So through the county, our leadership will be able to know ‘this area has this number of cutaneous* [leishmaniasis]*, and then they will pull the resources to that area so that they can deal with that condition*.

[Participant 15, community health nurse, level 2]

While not framed as formal design principles, these insights point to the importance of designing AI-embedded mHealth tools that are responsive to existing digital infrastructures and aligned with routine clinical workflows, rather than functioning as stand-alone applications. Further ethnographic work will enable more fine-grained articulation of how such integration is enacted in practice.

## Discussion

### Principal Findings

This study presents a comprehensive understanding of FHWs’ experience and perception of the WHO Skin NTDs app in Kenya. The majority of FHWs described the app as facilitating the following: (1) validating their clinical judgment, which reduced dependency on referral pathways and improved patient retention and community engagement; (2) incremental knowledge acquisition and growing confidence in managing dermatological conditions independently; and (3) enhanced patient engagement and perceived trust in the diagnostic process.

These findings contribute to a growing body of evidence on how mHealth tools are being integrated into resource-limited frontline health systems and the ethical and pedagogical challenges this raises, particularly given the app’s intended function as an educational and capacity-building tool [21,30,40]. By contrasting FHWs perceptions of pre- and postapp clinical workflows (Figures 3 and 4) and integrating FHW narratives (Multimedia Appendix 3) with thematic analysis, we identify potential system-level challenges associated with dermatological care and specific mechanisms of change facilitated by the app.

These insights inform both app refinement and broader implementation strategies to maximize pedagogical value and ensure ethical integration of mHealth tools in frontline health care systems. Taken together, they suggest that the app is having a positive impact on dermatological care within the studied context and is, in many cases, being used in alignment with its educational remit. However, there remains a need to examine edge use cases and potential risks—particularly where usage may extend beyond the app’s original design parameters [23].

### Clinical Improvisation and AI-Supported Reasoning

This study highlights the central role of clinical improvisation—real-time adaptation of clinical workflows and care strategies—as a core component of dermatological care in resource-limited settings [41,42]. In sub-Saharan Africa, FHWs routinely navigate complex social, political, infrastructural, and economic constraints, necessitating clinical improvisation [41]. FHWs identified multiple systemic barriers impacting dermatological care, including limited diagnostic resources, inadequate training, scarce dermatology expertise, and high patient volumes (Figure 3). In response, they described improvisational practices such as habitual referral, informal WhatsApp consultations, and public deliberation of clinical cases (Figure 3). Crucially, these practices are not merely indicative of knowledge gaps or health system limitations; rather, they reflect locally adapted strategies developed under constraints, described elsewhere as the “craft” of care under constraint [41].

However, while such strategies may enable care delivery where formal systems fall short, our findings suggest that some may also inadvertently limit opportunities for building and maintaining dermatological knowledge and case management skills. In this study, FHWs linked habitual referral and reliance on external consultation to diagnostic delays, patient attrition, and stigma–patterns observed across similar African contexts [43,44]. These findings suggest that some adaptive strategies may unintentionally reinforce ongoing dependency on specialist services by reducing opportunities for local dermatological knowledge development and case management experience.

Our findings suggest that integrating the WHO Skin NTDs app into clinical workflows may help reshape improvisational practices by facilitating more reflective diagnostic reasoning and greater confidence in local facility case management. While some of these benefits may also arise through other forms of clinical decision support, the AI-enabled nature of the app allows FHWs to compare image-based differential diagnoses with their own clinical assessments in real time.

In the broader literature, AI-embedded tools are ideally situated within a model of human-AI symbiosis: a collaborative relationship where the clinician’s reasoning complements the algorithm’s suggestions [45,46]. Most FHWs in our study described using the app in these terms—referring to it as a “partner” [Participant 15], akin to a second reader that supports and moderates, rather than supplants, their clinical judgment. FHWs reflected that this perceived partnership enabled more confident and independent patient management at the local-level facility, reducing reliance on improvisational workarounds such as habitual referral. This could be interpreted through the lens of self-efficacy theory, which posits that an individual’s engagement in a behavior is related to their belief in their ability to perform such tasks [47]. In this context, the app appears to function as a mechanism for enhancing FHWs’ perceived self-efficacy in dermatological care, supporting their ability to act on clinical decisions locally. This reflects wider literature on how AI-embedded clinical decision support systems can be, and should be, designed to augment rather than replace human reasoning [46,48].

However, our findings also suggest that when the app’s output did not align with the FHW’s clinical judgment, FHWs often reverted to referral to specialist dermatologists. While this can be understood as an appropriate response to clinical uncertainty, it also highlights an important opportunity for future design refinement. AI-embedded decision support tools may benefit from incorporating structured support mechanisms to guide arbitration in such cases, ensuring that the burden of resolving diagnostic discrepancies does not fall solely on the FHW. Such mechanisms could further enhance the app’s pedagogical value by facilitating reflection and learning through structured resolution pathways, rather than relying on referral as a default form of risk mitigation.

### Ethical Tensions: Maintaining Clinical Autonomy in AI-Supported Care

Our study raises a potential ethical concern with respect to how some FHWs may inadvertently use the app beyond its intended remit as an educational clinical decision support tool, potentially engaging in care beyond their clinical competence [23]. One FHW, for example, explicitly described deferring entirely to the app’s diagnostic suggestions. This pattern may reflect elements of automation bias, whereby users place undue trust in algorithmic outputs over their own clinical judgment [49]. Given the limited dermatological knowledge reported by FHWs, such potential overreliance may raise medicolegal and ethical concerns [23].

Some scholars caution that as AI systems are perceived to outperform human judgment, they may begin to displace human epistemic authority [50]. This risk is echoed in broader literature, which highlights that AI tools may contribute to clinician deskilling, diminished critical reasoning, and reduced capacity to navigate complex or atypical cases through overreliance [48-53]. While often discussed as a future-facing concern, our findings suggest that elements of this dynamic may already be emerging in contexts where dermatological expertise is limited [50]. In such cases, for some FHWs, the app’s output may be seen not as suggestive but as directive—an inversion of its intended role.

Meaningful human-AI collaboration depends on users having sufficient knowledge to question and validate algorithmic suggestions—a process that relies on training, confidence, and the time and institutional space to exercise clinical judgment [46]. Many FHWs in our study described using the app in this way, comparing AI-generated differentials with their own clinical assessments and reporting perceived knowledge gains through continued app use. Such experiences may, over time, strengthen their capacity to engage critically with AI outputs. Conversely, where foundational dermatological knowledge is limited, the same system may increase the risk of inappropriate reliance on algorithmic suggestions.

This presents a paradox in the positioning of the WHO Skin NTD app. The tool is explicitly designed to support capacity building among FHWs in response to limited dermatological expertise [21,22,30]. Yet, for the app to be used responsibly, FHWs must possess sufficient baseline knowledge to critically interrogate and contextualize its outputs. As our results suggest, this prerequisite cannot be assumed and is likely to vary significantly across different health system contexts, facility levels, and individual FHWs. To help mitigate this risk, explainability of AI outputs is critical: presenting outputs in transparent, interpretable ways can better support FHWs in understanding and validating diagnostic suggestions [54]. Other human-centered design principles, such as offering contextual guidance and supporting stepwise reasoning, could further assist FHWs in maintaining epistemic authority while engaging with the app. Features already present in the app, such as graded confidence levels associated with differentials, may help surface diagnostic uncertainty in ways that encourage critical engagement [54-56].

In addition to this epistemic tension, it is critical to recognize that the dynamics of AI use in clinical practice are not shaped by individual knowledge alone. Broader contextual factors, including patient load, time constraints, and infrastructural limitations, also influence how AI outputs are interpreted and acted upon [45]. This reinforces the importance of designing and adapting AI tools and implementation strategies in ways that explicitly protect and promote clinical autonomy, both in individual decision-making and across broader health care delivery [45,57,58].

Three areas of further work are needed. First, direct observation of app use in situ will be critical to understanding how it is conceptualized in practice, how broader contextual factors shape its use and whether it is functioning as intended (as a clinical decision support tool used for educational purposes within the clinical encounter), or whether some usage patterns risk reclassification. This will also be critical for elucidating how patients perceive the app's role in the collaborative context of FHW-patient interaction. Second, targeted design adaptations, such as embedded disclaimers or periodic training modules, should be explored to reinforce the app’s role as a nondirective support tool and bolster FHWs’ dermatological knowledge to safeguard clinical autonomy. Third, development of a context-specific professional ethical guidance framework for health care AI use (PEG-AI) may be necessary to ensure the safe, responsible, and ethically robust integration of the app into frontline care [23]. PEG-AI offers a context-sensitive governance approach for AI-embedded tools, essential for ensuring ethical implementation in resource-limited settings and safeguarding clinical autonomy, regardless of medical device classification [23].

Maintaining the use of the app within the boundaries of clinical competencies is therefore not solely a function of the tool itself but depends on the interaction between design features (eg, explainability, confidence indicators, and embedded guidance) and broader system-level supports, including training, workflow integration, and context-specific ethical governance.

### Situated Learning

In our study, only 1 FHW had ever heard of the WHO manual “Recognising Neglected Tropical Diseases Through Changes on the Skin: A Training Guide for Frontline Health Workers” [5]—a training guide specifically developed to build dermatological capacity among FHWs in resource-limited settings. Interestingly, the entire contents of the app are derived from this manual. However, our findings suggest that the format in which the information is both accessed and delivered shapes engagement and learning. While the manual offers comprehensive guidance, its didactic, decontextualized format perhaps separates learning from real-world clinical application. By contrast, the app delivers this same content in ways that are tailored to individual clinical cases, offering real-time feedback and follow-up information that are directly tied to patient encounters. Embedded in the relational space of the consultation, the app appears to support experiential, situated learning [59]. This aligns with well-established principles from the situated learning literature which emphasizes that learning is most effective when embedded within authentic contexts of practice [60-62].

In optimal conditions, the affordance of a continuously connected mobile device likely enhances this mode of learning. However, in our study, limited internet connectivity emerged as a barrier to consistent app use. Ensuring that the app’s AI algorithm functions offline will be essential for equitable and sustained integration—an improvement the WHO Department for NTDs is currently exploring.

### System-Level Impact and Referral Pathway

Existing literature suggests that over 80% of dermatological cases encountered by FHWs at the primary care level are common skin conditions, which are generally simpler to diagnose and manage [31]. In our study, FHWs reported that the app supported their ability to identify and manage these common skin conditions, often confirming their clinical judgment and enabling local facility management. However, when confronted with complex cases—particularly suspected skin NTDs—our findings show that FHWs remained more likely to defer to specialist referral, especially when the app’s diagnostic output could not be corroborated by their own limited dermatological knowledge. This pattern aligns with a key dimension of the PEG-AI framework which emphasizes that AI-embedded tools should be used within the boundaries of clinical competencies [23].

In this context, the WHO Skin NTDs app appears to strengthen local dermatological care by enabling FHWs to manage common skin conditions more confidently, potentially reducing unnecessary referrals. This may help alleviate pressure on overburdened dermatology services, allowing specialists to focus on more complex or rare presentations, particularly skin NTDs.

However, this shift in clinical workflow introduces algorithmic implications. As FHWs increasingly retain patients at the facility level, the accuracy of the algorithm becomes more consequential. In such cases, it may be preferable for the algorithm to prioritize sensitivity over specificity (ie, to favor false positives over false negatives) to minimize the risk of missed cases, particularly as reliance on referral as a default pathway has declined (Figures 3 and 4).

This shift also raises important system-level considerations. While strengthening that facility-level management may improve access to care and reduce pressure on referral systems, it may also introduce new risks. If only suspected skin NTDs cases are escalated—while structural barriers to referral (such as distance, cost, and dermatologist shortages) remain—patients with complex dermatological conditions may still face delayed diagnosis and discontinuity of care, particularly in rural and underserved areas.

Assessing the full impact of AI-embedded tools such as the WHO Skin NTDs app—both intended and unintended—requires moving beyond the individual clinical encounter to examine how such tools reshape referral pathways, clinical workflows, and health system capacity. A systems-level process evaluation will be essential to understand these dynamics and their implications for patient outcomes, including health economic considerations [63,64]. This aligns with broader calls to evaluate mHealth interventions not only at the point of care but across the full health system to surface both enabling factors and unintended consequences [65].

### Illness Trajectories

A key challenge frequently reported by FHWs was the lack of immediate management and follow-up support following the app's diagnostic suggestions. While the app provides AI-generated diagnostic suggestions instantaneously, the accompanying disease management guidance—although present—is in a separate section on the app and not seamlessly integrated into the clinical decision support workflow. This design choice may inadvertently reinforce a fragmented view of care, privileging diagnosis as an endpoint rather than as part of a broader continuum that includes, for example, management, follow-up, and psychosocial support [41]. This is particularly relevant, given the chronic course and complex illness trajectories associated with many common skin conditions and skin NTDs [66].

Enhancing the app’s capacity to comprehensively support FHWs across the full illness trajectory—by embedding management guidance directly after diagnostic suggestion—could significantly broaden its value. The section of the app describing how to diagnose and treat each skin disease incorporates links to targeted training modules or embedded decision pathways (such as those aligned with WHO Academy resources) to better equip FHWs to deliver holistic, sustained care that responds to the complex and chronic illness trajectories of dermatological conditions [67].

### Limitations

This study has several limitations. First, findings reflect the perspectives of FHWs only; patient perspectives on the app’s influence on care, agency, and experience were not explored. This is a key aim that will be addressed in forthcoming ethnographic fieldwork. Second, FHW responses may have been influenced by social desirability bias, given some of the research team’s involvement with the app’s development. However, independent facilitation was used to mitigate this risk [68,69]. Third, reported shifts in care may reflect short-term novelty effects rather than sustained practice change [70].

While this study aimed to generate a systems-level understanding of the app’s implementation, future research should complement this with detailed patient pathway mapping and comparative studies across diverse contexts to assess transferability [62]. Of the 50 FHWs initially enrolled, 47 participated in the training and deployment phase, and 36 FHWs attended the follow-up workshop where qualitative data collection occurred (72% follow-up). This is notable, given the wide geographical spread of participants and the logistical barriers involved in attending a centralized workshop. All 5 counties were represented among those who attended. However, it is possible that nonattendees had lower engagement with the app during the deployment phase, although no definitive reasons were provided for their absence. This potential attrition bias should be considered when interpreting findings.

### Conclusions

This study offers preliminary insights into the ways FHWs perceive the AI component of the WHO Skin NTDs app is reshaping frontline dermatological care in a complex, resource-limited health system. FHWs reported that the app supported more confident local-level facility management, disrupted habitual referral patterns, and fostered incremental knowledge acquisition. Importantly, our findings highlight the ethical and pedagogical complexities of integrating AI-embedded tools into frontline care. While FHWs reported that the app often strengthened clinical reasoning and learning, risks of overreliance and unintended shifts in clinical autonomy remain, particularly where baseline dermatological knowledge is limited. Future research should explore these dynamics through in situ observation and pathway-level evaluations. With continued attention to both pedagogical value and ethical governance, the WHO Skin NTD app has strong potential to advance the WHO’s vision of equitable, person-centered skin health for all.

## Supplementary material

10.2196/81829 Multimedia Appendix 1 Semistructured interview topic guide.

10.2196/81829 Multimedia Appendix 2 Focus group discussion topic guide.

10.2196/81829 Multimedia Appendix 3 Supporting quotes for causal loop diagrams.

10.2196/81829 Checklist 1 COREQ (Consolidated Criteria for Reporting Qualitative Research).

## Abbreviations

AI: artificial intelligence
CLD: causal loop diagram
EMR: electronic medical record
FGD: focus group discussion
FHW: frontline health worker
KEMRI: Kenyan Medical Research Institute
mHealth: mobile health
NTD: Neglected Tropical Disease
PEG-AI: professional ethical guidance framework for health care AI use
SSI: semistructured interview
WHO: World Health Organization
