# Supplementary material for: Frontline Health Workers’ Perspectives of the World Health Organization Skin Neglected Tropical Diseases App in Kenya: Qualitative Study on AI-Embedded mHealth Implementation
Source: JMIR Mhealth Uhealth. 2026 Jul 14;14:e81829. doi: 10.2196/81829 (PMC13367759; doi:10.2196/81829)
Supplement: Multimedia Appendix 1 [file mhealth-v14-e81829-s001.docx]

**Multimedia Appendix 1.** Semistructured interview topic guide.

**Introduction**

Hi {interviewee’s name}, I’m {interviewer’s name}. Thank you for taking the time share your thoughts surrounding the WHO skin NTD app and your experience using it.

Are you okay for us to start recording? I will now begin the recording.

Today, we'll explore your use of the WHO skin NTD app as a diagnostic support and training tool for skin NTDs and common skin conditions. There are no right or wrong answers here; this is an open space for you to share your views and experiences. Your insights are incredibly valuable. They'll help us better understand how the WHO skin NTD can support FHWs in sub-Saharan Africa. If during the interview, you would like a break or you would like to stop completely, please let me know and we can do so.

Before we start the interview, how does that sound to you? Do you have any questions?

**Contextual Opening:**

1. Can you describe the healthcare facility where you work, your dermatological training/ experience and the types of patients you usually see?

*(Focus: Factual information about facility and personal background)*

1. Could you walk us through your usual process for diagnosing a skin NTD or common skin condition? This can include any tests you use, decision-making steps, available equipment, and how you communicate with patients during diagnosis.

*(Focus: Step-by-step overview of current diagnostic practices from the point of patient arrival to completion of treatment, capturing all elements of the diagnostic journey)*

1. What are the current barriers or challenges you face within this diagnostic pathway?

*(Follow-up: Explore specific challenges, such as resource limitations, equipment shortages, or communication issues with patients)*

1. Do you or your colleagues typically use health apps in practice? Which ones? What’s your view on using technology like health apps for managing specific conditions?

**Use Case Exploration of the WHO skin NTD app:**

1. How long and how often have you used the app? What’s your overall opinion of it?
2. Could you describe a specific case when you used the WHO Skin NTD app with a patient?
3. What steps did you take to reach a diagnosis in that instance? How did the app influence or adjust your typical diagnostic process?
4. Which features of the app were particularly useful in that case? Are there features that you found less useful? Why?
5. How do you think using the app affected the patient’s experience or outcome? Did it change the way you communicated with or involved the patient in the diagnostic process?
6. Reflecting on your experience from the start of using the app, how has it impacted your confidence in diagnosing skin NTDs and common skin conditions?
7. Were you aware of the WHO manual “Recognizing neglected tropical diseases through changes on the skin: a training guide for front-line health workers” published 6 years ago? [Recognizing neglected tropical diseases through changes on the skin A training guide for front-line health workers](https://www.who.int/publications/i/item/9789241513531)

1. If so, did you receive a hard copy of that manual?

1. Would you recommend the WHO skin NTDs app for it to be used country wide in a routine way as an educational tool?
2. Do you see the app being incorporated as a standard medical device in your workplace? Why or why not?

*(Focus: Understanding how the app fits into the diagnostic workflow and its impact on patient interaction.)*

**Closing**

Thank you for your insights. Your experience provides invaluable context to our research and will help shape the future development and implementation of tools like the WHO Skin NTD app. Is there anything else anyone would like to add or clarify based on our conversation today?
