# Supplementary material for: Frontline Health Workers’ Perspectives of the World Health Organization Skin Neglected Tropical Diseases App in Kenya: Qualitative Study on AI-Embedded mHealth Implementation
Source: JMIR Mhealth Uhealth. 2026 Jul 14;14:e81829. doi: 10.2196/81829 (PMC13367759; doi:10.2196/81829)
Supplement: Multimedia Appendix 2 [file mhealth-v14-e81829-s002.docx]

**Multimedia Appendix 2.** Focus group topic guide (4 focus groups, 1.5 hours each).

**Introduction**

Welcome, everyone. I’m {facilitator’s name} and this is {facilitator’s name}. Thank you all for joining today’s discussion on the WHO Skin NTD app. Your shared experiences and thoughts are valuable to us, as they provide a collective view of how the app is being used in real healthcare settings.

There are no right or wrong answers here; we’re interested in hearing each of your perspectives. Feel free to add to each other’s points or to share different viewpoints – all are valid. This is an open and safe space to discuss your experiences, and all responses will be anonymised.

If at any point you’d like to pause or step out, please let us know. Is everyone comfortable with beginning the recording?

**Contextual opening**

1. We realise that you may not know each other well, so to start, could each of you briefly introduce yourselves, your role, and how long you’ve been working with skin NTDs and other common skin conditions in your facility?

**Group Reflections on App Use**

1. Let’s begin with your first impressions of the WHO Skin NTD app. What were some of your initial thoughts or expectations when you first started using it, and did any of these change over time? How often did you use the app?
2. From your experience, what kinds of cases seem to benefit the most from using the app? Are there types of cases where the app seems less effective or helpful?

**Impact of the App on Workflow:**

1. Has the WHO Skin NTD app influenced your approach to diagnosing skin conditions? In what ways?
2. Overall, has using the app saved you time or added complexity to your work? Could you share some specific examples?

**Knowledge Gains:**

1. What have you learned about skin NTDs and other common skin conditions through using the app?
2. How has the differential diagnosis provided by the app influenced your understanding of these diseases?
3. Are there insights or skills you’ve gained from using the app that you now apply outside of app use?

**Reflection on Challenges:**

1. What practical challenges or barriers have you encountered when using the app in your specific healthcare setting?

*(Focus: Identify practical challenges, such as connectivity issues, lack of familiarity with the app, or compatibility with existing processes.)*

1. How did you manage situations when the app’s suggestions (differential diagnosis) didn’t align with your own expectations or clinical judgment? What strategies did you use to manage any differences?

**Future Use and Recommendations:**

1. Do you see yourself continuing to use the app in the future? Why or why not?
2. What changes or additions would make the app more useful for your work?
3. What features must the app have for you to use it or recommend it to someone?
4. What do you think would be the best way to promote and encourage the use of this app among FHWs like yourself? Which organisations should be involved in the dissemination of the app?
5. Do you see the app being incorporated as a standard medical device in your workplace? Why or why not?

*(Focus: Suggestions for app dissemination and adoption strategies)*

**Closing**

Thank you all for your insights. Your experiences provide invaluable context to our research and will help shape the future development of tools like the WHO Skin NTD app. Is there anything else anyone would like to add?
