# Supplementary material for: Frontline Health Workers’ Perspectives of the World Health Organization Skin Neglected Tropical Diseases App in Kenya: Qualitative Study on AI-Embedded mHealth Implementation
Source: JMIR Mhealth Uhealth. 2026 Jul 14;14:e81829. doi: 10.2196/81829 (PMC13367759; doi:10.2196/81829)
Supplement: Multimedia Appendix 3 [file mhealth-v14-e81829-s003.docx]

**Multimedia Appendix 3**

| **CLD 1 - pre-app context, systemic challenges in dermatological care** | | | |
| --- | --- | --- | --- |
| **Diagram element** | **Theme** | **Supporting quotes** | **Participant (ID)** |
| Lack of dermatological knowledge | 2 | For myself, I think I have very little knowledge about skin or maybe dermatology. Because I am a clinician, I have never specialised in skin diseases.    Before the app was introduced we had barriers like knowledge gap of how to diagnose and how to manage skin diseases. | Ppt 24          Ppt 21 |
| Lack of dermatological training | 1 | In our facility, we are two clinicians, 2 nurses.. We don't have dermatologists. No one has been trained about dermatology.    So my dermatological experience is basically the the basic. I'm not trained dermatologist. So it's the basic knowledge which I required through when I attended medical school. And the condition around are wide and in case we we are not able to provide the services, the dermatological services, we usually refer them to a dermatologist. | Ppt 23          Ppt 19 |
| Lack of access to laboratory diagnostic resources | 1 | We don't have the lab, the lab test for testing the skin diseases.    Actually we are lacking so much in our facility, like the lab works, even the baseline ones, it becomes a challenge, a very big challenge, so we find ourselves just leaning on clinical diagnosis and then referring the patients to a dermatologist. | Ppt 1      Ppt 18 |
| Strain/ pressure on dermatologists | 1 | We have very few dermatologists in our facilities. Maybe only in the county referral hospitals… I was even talking to the clinicians working in the dermatology department and they were saying ‘most of us we are due to retire soon. And we are worried about the skin conditions and how they can be managed’.    We have few, the dermatologists are few…So if you see that the ratio between the patient and the dermatologist, the patient are so many compared to the to the dermatologist…So you'll find that the patient will not get the services…they need to wait for a long time until the dermatologist come again and then.. to come and review the patient. So the challenge we have is the human resource. | Ppt 36                Ppt 1 |
| Diagnostic delay/ loss to FU | 1 | And when we tried to attach them to the facility, in fact they refuse. And there were so many defaulters.    From my experience I also felt that most of these skin conditions are managed for quite a long duration of time, and actually referring these cases from the rural facilities, and going to the county hospitals, most of the clients don’t even go. They lack transport. They lack means of reaching to the facility. | Ppt 2        Ppt 36 |
| Habitual referral | 1 | The patient with skin NTDs... we didn't want much to know much about it since we didn't know how to make the diagnosis... we didn't want to learn. Because we just felt like, OK, it is. It was difficult to make diagnosis and in terms of management, it was still difficult for us to manage those conditions… we were not in a position to make that diagnosis. We were taking it as a part of specialised. These were conditions that were supposed to be managed by specialist. Specialised people, not by us. Yeah, ours was clearing [patients] and referring… and we wouldn’t have followed up. | Ppt 3 |
| Clinical detachment from dermatological cases | 1 | Normally I used to refer patients and I tell them, come on Thursday, that's when the dermatologist will come and maybe we'll get treatment.    In our outpatient department… initially whenever you used to see any skin condition regardless we we used just to refer. It is clearly not for me. You don't want even to waste the time with it…it is clearing and referring… We didn't want to learn…. there was no need for us to learn about skin NTDs because we didn't, we didn't see any interests…since we were not in a position to make that diagnosis. I I can. OK. We were taking it as a part of specialised. These were conditions that were supposed to be managed by specialist. Specialised people, not by us. Yeah, ours was clearing [patients] and referring. Just refer. | Ppt 9          Ppt 3 |
| Patient reduction in confidence in FHW clinical ability | 1 | Yeah, if a patient comes in and you are not sure of the diagnosis, the patient may feel that this clinician is not confident. ‘Why has the clinician have to call others to come and verify the diagnosis, it means that the the the clinician is not confident enough…. even the even the doctor, even the clinician doesn't know what I'm suffering from’. They will not even feel confident with the treatment that you write for them.    You inquire from dermatologist and maybe that person is not reachable. So it is like, you feel sometimes that you've not done something good today because of the delay. And they [the patient] will just see you as this clinician is like he or she doesn't know anything concerning this condition. | Ppt 2                  Ppt 11 |
| Stigma | 2 | The [patients] come to the facility and then you find a clinician, the clinician is alone. They may be not very sure if this is leishmaniasis or not leishmaniasis, so they  call each other, you have to call each other. Even from the office where we had the clinic, the facility….You see by the time you are calling more people to come and see you see, the patient feels stigmatised.      In the community, because we don't have a specialist for skin NTDs… when you are out there [in the community], if there is no person who knows about cutaneous, that patient earlier [before the app] was being referred. And sometimes when you tell the patient to come from where you went for outreach, they may not come. Because.. they are stigmatised. ‘Why am I being told this, there are other patients that are being treated after going to the facility, me, I am being referred so far to the health facility... | Ppt 2                    Ppt 15 |
| **CLD 2 - post-app integration, shifts in clinical practice and health system dynamics** | | | |
| **Diagram element** | **Theme** | **Supporting quotes** | **Participant (ID)** |
| Improved dermatological knowledge | 2 | The app challenged me also about my knowledge of skin diseases. When it brought me a certain diagnosis that I didn’t know before, I also had very much desire to go and read from the books, to understand more about the disease.    The app has impressed me to research on more conditions…. maybe you think that it was scabies. It [the app differential diagnosis] has brought another condition that you have never seen, you have never known. So you go to inquire more about that condition and you compare, maybe what you have seen in other conditions, clinical manifestations compared to that app.    When we got this app. One thing it helps you to study more, because at least after seeing the diagnosis, at least you have to read from the Google that is or any other platform about leishmaniasis. Any condition that you have been given by the by the app you end up reading on it. So at least you equip yourself with knowledge and management, which is an advantage.    I was curious to know more about those condition that I didn't know. So I kept on going back to books and to date I am still like that. | Ppt 33          Ppt 14          Ppt 3            Ppt 32 |
| Clinical ownership of dermatological cases | 1 | I shared with some colleagues that if maybe you get any skin conditions, you can tell me.    From the time we started using this app, it has, really had a positive impact. Because in our health facility, if anyone comes across a patient with skin lesion, he or she will first send the patient to me. Even if I am off, they will call me.. ‘come and see there is a patient with a skin lesion, come and use the app, we see what is the problem’.    In the outpatient department, I was only the only one who had this app, the clinical officer. So I was telling my colleagues in case you see this case, you can always call me, then we can do something, then we'll see whether this is correct or not. So, in many cases, currently it's like if they see anything, just call ppt 13, let him come and see.    Currently in outpatient Department, in fact, I tell them to bring skin conditions into my room… I just want to read, to see them [skin patients] and to read. And by the end of the day, it has helped me to improve my skills in the management of common skin conditions and skin NTD… but right now at least you have the skills to examine. | Ppt 24      Ppt 21                Ppt 13        Ppt 3 |
| Proactive management at facility level | 1 | I did not refer them as I used to refer at first… I was calling the dermatologist and I had even something to discuss with the dermatologist.    We used to refer all patients with skin conditions. But now with the app, we try to manage the patients without sending all of them to the dermatologist | Ppt 9        Ppt 32 |
| Improved confidence in managing dermatological cases | 2 | With this app you with those… there's some things that you can learn when you take an image and then get the condition you can go and read [about the differential diagnosis], so there’s that confidence building up.    My side yeah, I think owning the app, because I was the only person on the app in the facility, it gave me confidence to see many patients who have skin diseases. I was also very much encouraged to see patients who have skin diseases since I have confidence that I will manage to use the app to differentiate to know which type of disease is this. And the app really helped me to diagnose most of these skin diseases…. I work I work in a rural facility where we don't have a dermatologist, but now owning the app it really helped me to have confidence.  So it [the app] has impacted me positively because now I'm very confident to tackle any skin condition rather than before. Before seeing that patient, you can see the patient from far and then you are thinking me how am I going to manage this patient now. How can where can I start, from what you see? But when this app came, you have that confidence to see that patient and come up with a diagnosis. | Ppt 19         Ppt 34 |
| Destigmatisation of skin conditions | 2 | Before we get hold of this app, when you see a condition where you suspect this is an NTD, you have to call your colleague, maybe even 2-3 people to come on to come to the same room and discuss the patient. So for now at least there is that reduction in the stigma because you are only with a patient.  So there is a lot of confidentiality and they have more confidence and be able to treat your patient earlier. | Ppt 13 |
| Patient impact – early diagnosis and treatment | 1 | It [WHO skin NTD app] is a key app for any primary healthcare worker, because one, it's it's it's going to help in giving the treatment to the patients… it's going to stop delay in giving the patient treatment. And that will reduce the complication associated with each particular condition depending on each condition you are dealing with. Cause as we delay there are problems of that condition. | Ppt 18 |
| Patient confidence | 1 | I was attending a client…condition which is chronic...he had some lesions on the face… we took the the I took the photo, I uploaded and then the app gave me the results and then the process I tried to manage though I told him we need to to to review regularly. So in the process of reviewing him and the management I gave him, and I think there's some **i**mprovement in the confidence 'cause when he comes to that facility, he'll always ask for me. Even if there are some other health workers 'cause there’s that bond. And I think he appreciated. | Ppt 19 |
| Patients as ambassadors to the community | 1 | And also to the community, once you use the app in diagnosing one patient, he or she will go on, referring another one, ‘go to the facility, the nurse will take a photo’…    Immediately when you take the photo, they [the patient] became automatically ambassadors to the community, then they [the community] come and look for it.    Another good thing is, after some patients had their photos, they go to other patients saying ‘there is an app, a skin disease app’. So they meet… and say there is someone who is doing a study, so more patients come.. some are still calling us saying ‘can you still do it, still diagnose with it?’ . | Ppt 29          Ppt 25    Ppt 1 |
