# Supplementary material for: Frontline Health Workers’ Perspectives of the World Health Organization Skin Neglected Tropical Diseases App in Kenya: Qualitative Study on AI-Embedded mHealth Implementation
Source: JMIR Mhealth Uhealth. 2026 Jul 14;14:e81829. doi: 10.2196/81829 (PMC13367759; doi:10.2196/81829)
Supplement: Checklist 1 [file mhealth-v14-e81829-s004.docx]

COREQ (Consolidated Criteria for Reporting Qualitative Research) checklist applied to:

**Frontline perspectives of the WHO skin NTD app in Kenya – a qualitative study on AI-infused mHealth implementation**

| **Topic** | | **Item No.** | | **Guide Questions/Description** | **Reported in section** |  |
| --- | --- | --- | --- | --- | --- | --- |
| **Domain 1: Research team**  **and reﬂexivity** | | | | | |  |
| *Personal characteristics* | | | | | |  |
| Interviewer/facilitator | | 1 | | Which author/s conducted the interview or focus group? | Methods: The interviewers (EQ, RN, CC, AF, DA) used semi-structured topic guides to guide FGDs and SSIs, which were reviewed by all authors prior to the workshop (see Appendix 3, 4).  Methods: Each FGD was co-facilitated by a local scientist from KEMRI (RN, DA) and a researcher from the Global North (EQ, CC). |  |
| Credentials | | 2 | | What were the researcher’s credentials? E.g. PhD, MD | The interview team consisted of five researchers with a range of academic credentials, including completed PhDs, PhDs in progress, and completed master’s degrees. The team included university professors, experienced research scientists, and doctoral researchers with expertise in public health and digital health.  At the time of the study, the researchers held a variety of academic and research positions, including doctoral researchers (EQ, AF), university lecturer (CC), senior research scientist at KEMRI (RN) and research assistant at KEMRI (DA).  All five interviewers were female. The broader research team involved in study design and data analysis included both male and female researchers. |  |
| Occupation | | 3 | | What was their occupation at the time of the study? |  |  |
| Gender | | 4 | | Was the researcher male or female? |  |  |
| Experience and training | | 5 | | What experience or training did the researcher have? | All interviewers had research methods and analysis training through post-graduate degree programmes and/or experience from prior global health research projects (2 - 15+ years of practice, each). |  |
| *Relationship with*  *participants* | | | | | |  |
| Relationship established | | 6 | | Was a relationship established prior to study commencement? | Author contributions: The participants did not have a professional or personal relationship with the researchers prior to the study. |  |
| Participant knowledge of  the interviewer | | 7 | | What did the participants know about the researcher? e.g. personal goals, reasons for doing the research | EK initially contacted all participants to explain the study’s objectives and potential impact, including its aim to explore the feasibility and integration of the WHO Skin NTD app in primary healthcare settings.  During the second workshop, the facilitation team (RN, DA, EQ, CC, AF) introduced themselves at the beginning of each FGD and SSI, outlining their professional backgrounds and personal motivations for engaging in this research |  |
| Interviewer characteristics | | 8 | | What characteristics were reported about the inter viewer/facilitator? e.g. Bias, assumptions, reasons and interests in the research topic | Methods: The interviewers (EQ, RN, CC, AF, DA) used topic guides to guide FGDs and SSIs which were reviewed by all authors prior to the workshop (see Appendix 3, 4).  Methods: All interviews and FGDs were conducted in English (an official language in Kenya). Each FGD was co-facilitated by a local scientist from KEMRI (RN, DA) and a researcher from the Global North (EQ, CC).  Author contributions: The participants did not have a professional or personal relationship with the researchers prior to the study.  The topic guides were designed to actively explore positive and negative perspectives of the WHO skin NTD app, equally. |  |
| **Domain 2: Study design** | | | | | |  |
| *Theoretical framework* | | | | | |  |
| Methodological orientation and Theory | | 9 | | What methodological orientation was stated to underpin the study? e.g. grounded theory, discourse analysis, ethnography, phenomenology,  content analysis | Methods: Given that this is the first qualitative assessment of the WHO Skin NTD app usability and acceptability, a bottom-up inductive coding approach was selected to support the iterative construction of themes grounded in participant narratives. This exploratory approach aimed to capture nuanced insights that may inform the application of appropriate theoretical frameworks in future studies, as the evidence base on the app’s integration expands. |  |
| *Participant selection* | | | | | |  |
| Sampling | | 10 | | How were participants selected? e.g. purposive, convenience,  consecutive, snowball | Methods: Five counties were purposively selected in collaboration with the Ministry of Health, Kenya. Within each county, health facilities and affiliated FHWs were identified with support from the County Departments of Health. From this sampling frame, 50 FHWs were randomly selected and invited by telephone to participate in an initial training and deployment workshop in April 2024. |  |
| Method of approach | | 11 | | How were participants approached? e.g. face-to-face, telephone, mail,  email |  |  |
| Sample size | | 12 | | How many participants were in the study? | Methods: Of the original 50 FHWs invited, 47 attended the first training workshop and participated in the deployment phase. Thirty-six attended the follow-up workshop, which included the main qualitative data collection activities. FGDs and SSIs were conducted during this second workshop to explore participants’ experiences using the app. |  |
| Non-participation | | 13 | | How many people refused to participate or dropped out? Reasons? | Methods: Of the original 50 FHWs invited, 47 attended the first training workshop and participated in the deployment phase. Thirty-six attended the follow-up workshop, which included the main qualitative data collection activities.  Limitations: This is notable given the wide geographical spread of participants and the logistical barriers involved in attending a centralised workshop. All five counties were represented among those who attended. However, it is possible non-attendees had lower engagement with the app during the deployment phase, although no definitive reasons were provided for their absence. This potential attrition bias should be considered when interpreting findings. |  |
| *Setting* | | | | | |  |
| Setting of data collection | | 14 | | Where was the data collected? e.g. home, clinic, workplace | Methods: The first workshop, held at a centrally located training centre in Kenya, introduced participants to the WHO Skin NTD app and provided training in its use. Participants were then invited to integrate the app into their routine clinical practice over a deployment period of approximately six months. Following this, all participating FHWs were invited to a second workshop (again, at a central location in Kenya) in November 2024 to reflect on their experiences using the app. |  |
| Presence of non-  participants | | 15 | | Was anyone else present besides the participants and researchers? | In the focus group discussions (FGDs), only the first author (EQ), co-authors (CC, DA, RN), and participants were present; no additional observers or non-participant individuals attended.  In the semi-structured (SSI) interviews, only the first author (EQ), co-authors (CC, DA, RN, AF), and participants were present; again, no additional individuals were present.  Methods: All interviews and FGDs were conducted in English (an official language in Kenya). Each FGD was co-facilitated by a local scientist from KEMRI (RN, DA) and a researcher from the Global North (EQ, CC). |  |
| Description of sample | | 16 | | What are the important characteristics of the sample? e.g. demographic  data, date | Methods: Participants were non-dermatologist FHWs who routinely manage patients with skin NTDs or common skin conditions at the primary care level…..50 FHWs were randomly selected and invited by telephone to participate in an initial training and deployment workshop in April 2024….The first workshop, held at a centrally located training centre in Kenya, introduced participants to the WHO Skin NTD app and provided training in its use. Participants were then invited to integrate the app into their routine clinical practice over a deployment period of approximately six months. Following this, all participating FHWs were invited to a second workshop in November 2024 to reflect on their experiences using the app.  Results: The 36 participants who attended the second workshop in November 2024 comprised FHWs from five counties in Kenya: Nakuru, n=11 (a fairly urbanised county situated in the Rift Valley); Baringo, n=8 (also situated in the Rift Valley, characterised by semi-urban and rural areas); Kajiado, n=6 (bordering Tanzania in southern Kenya with a semi-nomadic population); West Pokot, n=8 (a remote rural county bordering Uganda, predominantly inhabited by nomadic pastoralist communities) and Kwale, n=3 (a largely rural, coastal county in southeastern Kenya). (32–36) Participants included 14 Clinicians (Level 2 n=3, Level 4 n=4, Level 5 n=6, Coordinator n=1), 11 Clinical Officers (Level 3 n=2, Level 4 n=4, Level 5 n=5), and 7 Nurses (Level 2 n=4, Level 5 n=2, Coordinator n=1). Additionally, there were two Public Health Officers (Level 3 n=1, Level 5 n=1), one Doctor (Level 4), and one Pharmacy Technician (Level 3). Participant characteristics are described in Table 1. |  |
| *Data collection* | | | | | |  |
| Interview guide | | 17 | | Were questions, prompts, guides provided by the authors? Was it pilot tested? | Methods: The interviewers (EQ, RN, CC, AF, DA) used topic guides to guide FGDs and SSIs which were reviewed by all authors prior to the workshop (see Appendix 3, 4). |  |
| Repeat interviews | | 18 | | Were repeat inter views carried out? If yes, how many? | N/A, no repeat interviews were conducted. |  |
| Audio/visual recording | | 19 | | Did the research use audio or visual recording to collect the data? | Methods: Data collection and analysis. All focus group discussions and ss-interviews were recorded on portable Dictaphones and stored on a secured university-licensed cloud service (OneDrive). |  |
| Field notes | | 20 | | Were ﬁeld notes made during and/or after the interview or focus group? | Yes. FGDs were facilitated by two researchers, one who guided the discussion with the other documenting field notes.  Methods: Field notes were taken by all researchers during and after both FGDs and SSIs and were used to support the thematic analysis. |  |
| Duration | | 21 | | What was the duration of the inter views or focus group? | Methods (Data were collected through 15 semi-structured (SSI) interviews (each lasting 30 - 45 minutes) and four focus group discussions (FGDs) (1-1.5 hours, 8-10 FHW in each) |  |
| Data saturation | | 22 | | Was data saturation discussed? | Yes. However, in line with reflexive thematic analysis principles, the goal was not to achieve a finite saturation point but to develop a rich and nuanced understanding of FHW experiences across diverse healthcare contexts. |  |
| Transcripts returned | | 23 | | Were transcripts returned to participants for comment and/or correction? | No. Transcripts were not returned to participants for comment or correction, in line with the study protocol approved by Open University of Catalonia ethics, and to minimise participant burden. |  |
| **Domain 3: analysis and**  **ﬁndings** | | | | | | |
| *Data analysis* | | | | | | |
| Number of data coders | | 24 | | How many data coders coded the data? | | Methods: EQ conducted initial coding of all transcripts. A subset of transcripts was independently double-coded by AB and KO. Coding discrepancies were resolved through team discussion, and the codebook was iteratively refined. |
| Description of the coding  tree | | 25 | | Did authors provide a description of the coding tree? | | Yes. The coding structure was developed iteratively through reflexive thematic analysis. Initial codes were grouped into broader categories, which were then organised into higher-order themes and sub-themes, as presented in the Results section (Themes 1–4). The relationship between themes is also summarised in Figures 1 and 2. A full coding tree was not included in the paper due to space constraints, but a detailed coding hierarchy was developed and used to guide analysis. |
| Derivation of themes | | 26 | | Were themes identiﬁed in advance or derived from the data? | | Methods: Given that this is the first qualitative assessment of the AI component of the WHO Skin NTD app usability and acceptability, a bottom-up inductive coding approach was selected to support the iterative construction of themes grounded in participant narratives. This exploratory approach aimed to capture nuanced insights that may inform the application of appropriate theoretical frameworks in future studies, as the evidence base on the app’s integration expands. |
| Software | | 27 | | What software, if applicable, was used to manage the data? | | Methods: Audio recordings were transcribed verbatim by the lead author (EQ), supporting deep familiarisation with the data. Transcripts were analysed thematically using NVivo 15 software. |
| Participant checking | | 28 | | Did participants provide feedback on the ﬁndings? | | No. Participants did not provide feedback on the findings, in line with the study protocol approved by UoC ethics. This decision was made to minimise participant burden and in recognition of logistical constraints given the wide geographical spread of participants. However, findings were grounded through extensive iterative analysis, team-based reflexivity, and integration of verbatim participant narratives. |
| *Reporting* | | | | | | |
| Quotations presented | | 29 | | Were participant quotations presented to illustrate the themes/ﬁndings?  Was each quotation identiﬁed? e.g. participant number | | Yes, Results section provides illustrative quotes for each sub-theme with each quotation identified with the participant number. |
| Data and ﬁndings consistent | | 30 | | Was there consistency between the data presented and the ﬁndings? | | Results & Discussion section outlines consistency between data and findings. |
| Clarity of major themes | | 31 | | Were major themes clearly presented in the ﬁndings? | | Yes, Results section provides a descriptive explanation for each theme. |
| Clarity of minor themes | | 32 | | Is there a description of diverse cases or discussion of minor themes? | | Yes, Results section provides a descriptive explanation and illustrative quote for each theme and sub-theme. |
